# Review of human health exposure assessment frameworks in the United States and development of a unified framework for exposure assessment practitioners

**DOI:** 10.3389/fpubh.2026.1860994

**Published:** 2026-09-29

**Authors:** Melanie D. Nembhard, Corey L. Boles, Shannon H. Gaffney, Jason T. Lotter, Joshua R. Maskrey, Jennifer Sahmel

**Affiliations:** Insight Exposure and Risk Sciences Group, Boulder, CO, United States

**Keywords:** exposure assessment, exposure assessment framework, exposure assessment process, exposure science, harmonization, human health, risk assessment

## Abstract

The process of conducting a human health exposure assessment has been framed as a stepwise approach by multiple organizations and various authors. However, the recommended steps within these frameworks can vary depending on the organization or author referenced, and it is valuable to have a cohesive analysis of this important topic. The aim of this review was to systematically analyze and compile relevant exposure assessment frameworks in the United States (U.S.) and condense the differing recommended steps to create one consolidated, broadly applicable framework. A review of exposure assessment frameworks identified from U.S. governmental agencies, U.S. consensus guidance organizations, peer-reviewed scientific articles, and book chapters was performed. Inclusion and exclusion criteria were applied such that only comprehensive exposure assessment frameworks were included in the consolidation process. Five comprehensive exposure assessment frameworks from the U.S. Environmental Protection Agency, the American Industrial Hygiene Association, and the peer-reviewed scientific literature were determined to be appropriate for consolidation into the single framework. The steps identified within each existing approach were aligned to ensure all elements were incorporated into the creation of the final overarching framework, hereinafter referred to as the Unified Exposure Assessment Framework (UEAF) for human health risk assessment. The UEAF includes all essential elements of the identified published frameworks and further captures nuances within each source to allow practitioners to have one comprehensive process to reference when preparing to conduct an exposure assessment. Additional frameworks with specific nuances, or those identified as critical resources in the field, were also assessed and discussed. Through this effort, occupational and environmental health and safety (OEHS) and exposure science practitioners are provided with a consolidated crosswalk and proposed stepwise structure that may support more consistent consideration of key exposure assessment elements across U.S.-based occupational and environmental contexts.

## Introduction

Exposure assessment is a cornerstone of occupational and environmental health sciences and a critical component of the human health risk assessment process. As detailed by the National Research Council (NRC), the operating arm of the National Academies of Sciences, Engineering, and Medicine (NAS), in their seminal “Red Book” titled *Risk Assessment in the Federal Government: Managing the Process*, the significance of this process is that it provides a standard process for evaluation of risk that may be present from exposure to hazards (1). There are four essential steps encompassing the risk assessment process in the Red Book: hazard identification, dose–response assessment, exposure assessment, and risk characterization. These steps, in turn, inform risk management, which is a separate but highly interrelated process and provides methods for controlling exposures to a hazard to reduce or minimize the risk.

The risk assessment process provides a structured foundation for evaluating potential impacts to human health by integrating information about hazards, exposure conditions, and the potential likelihood and magnitude of resulting effects. Modern guidance emphasizes that risk assessment begins with planning, scoping, and problem formulation, during which decision contexts, assessment boundaries, and conceptual models are established before any quantitative work occurs (2–4). These foundational activities involve scientific judgment, stakeholder engagement, and transparent articulation of assumptions. Contemporary perspectives reinforce that risk assessment functions as a decision-support process, not a linear technical calculation, and therefore requires both analytical evaluations and structured non-analytical processes to ensure that decisions are scientifically grounded, proportionate, and transparent (5, 6). Within the analytical elements, current practice integrates evaluation of data quality, uncertainty, and variability, accompanied by clear communication of how these factors influence the ultimate interpretation of risk.

As a subset of the risk assessment process, the field of exposure science highlights exposure assessment as a core element that is necessary to provide information about the degree of contact with identified hazards. Guidance from the NRC describes exposure science as the study of the contact between stressors and receptors and the associated sources, pathways, and processes that influence potential health effects (7). This perspective establishes exposure science as the basis for understanding how chemical, physical, biological, and other relevant agents or stressors reach humans across environmental and occupational settings. Exposure science encompasses both quantitative and qualitative characterizations of exposure, including the magnitude, frequency, duration, and pathways of contact, and integrates these results across diverse data streams, such as from empirical measurements or modeling efforts (3, 7, 8). Contemporary international efforts similarly describe exposure science as a systems-oriented discipline that combines conceptual model development, pathway analysis, and evaluation of exposure determinants to link real-world conditions with potential health outcomes (9, 10). Because exposure assessment operationalizes these concepts within risk assessment, exposure science plays a central role in regulatory decision-making, workplace risk management, and the broader scientific understanding of how exposures influence human health effects. Recent advances in biomonitoring, computational exposure science, high-throughput and pathway-based approaches, and life-cycle perspectives further demonstrate the expanding importance of exposure science in supporting scientifically-grounded risk assessments (7, 9–11).

Establishing systematic and consistent approaches for conducting exposure assessments has been a longstanding priority across regulatory, scientific, and professional exposure science communities in the United States (U.S.) and beyond. In both occupational and environmental settings, human health exposure assessment supports diverse objectives: ensuring compliance with regulatory standards, identifying the need for controls, investigating potential health complaints, conducting retrospective exposure assessments or reconstructions, and supporting epidemiological research. These diverse applications and importance to workers and community health underscore the need for an organized and transparent exposure assessment process. To help guide occupational and environmental health and safety (OEHS) and exposure science practitioners, a number of organizations and authors have described exposure assessment as a structured, stepwise framework. Even when formal processes or procedures are not specified, the steps identified across entities and authors generally direct practitioners through many of the same underlying concepts.

Despite the many shared elements among the varying processes, some notable differences exist with respect to terminology, emphasis, and even specific steps identified as necessary components of the process. For example, some frameworks prioritize problem formulation and scoping, while others emphasize data analysis or communication. Terminological inconsistencies, such as whether to define a “similar exposure group” (SEG), or not, can create confusion among practitioners and variability in the exposure assessments performed by practitioners. Moreover, not all frameworks explicitly address steps such as goal setting, validation, or routine reassessment or confirmation. Additionally, many documents exist with general or partial approaches to, or elements of exposure assessment (e.g., a focus on steps for exposure pathway evaluation, or tools for use when evaluating exposure). This body of literature provides an unwieldy amount of information for the typical practitioner to process and evaluate in order to determine the best methodology for performing an exposure assessment. To address these challenges, a narrative review was performed to harmonize the major U.S. exposure assessment processes into a single consolidated framework for the OEHS practitioner.

Although international processes and frameworks for exposure assessment exist, these may be designed to address exposure assessments within different regulatory structures or risk assessment approaches. Additionally, the differences in methodological emphasis, approaches to hazard and risk characterization, and the degree of regulatory integration between the U.S. and, specifically, European regulatory contexts highlight some of the challenges of synthesizing and combining all international frameworks within a single structure. Therefore, for purposes of this review, international frameworks were excluded from the current analysis.

Based on this analysis, a novel overarching framework, the Unified Exposure Assessment Framework (UEAF), was developed. The UEAF presents a consolidated process that integrates and maintains essential elements from identified approaches in the U.S. The UEAF’s design focuses on providing clarity, transparency, and consistency to practitioners while maintaining flexibility for diverse applications in both occupational and environmental human health exposure domains. Through this effort, the goal was to equip OEHS and exposure science practitioners with a clear and repeatable framework containing a consistent stepwise process that reduces ambiguity, enhances comparability, and strengthens the foundation for robust, reliable, and thorough exposure assessments in the U.S.

## Methods

To identify established human health exposure assessment approaches in the U.S., this review systematically examined regulatory agency guidance, consensus organization documentation (including books, standards, technical manuals, and organization webpages), and human health exposure assessment frameworks described in peer-reviewed scientific articles and book chapters. For the purposes of this analysis, the term “framework” was applied broadly to any identified structured sequence of steps or methods that provided a clear and comprehensive process for conducting an exposure assessment, regardless of whether it was explicitly labeled as such. To be considered clear, the framework needed to provide a stepwise process to follow for an exposure assessment. To be considered comprehensive, the first step identified in the framework needed to establish the reason for the assessment, which aligned with problem formulation, otherwise referred to as goal setting or scoping, and one of the final steps identified needed to involve communication to stakeholders and/or documentation of the assessment.

Frameworks from agencies and consensus organizations were eligible for inclusion in the comparative analysis if the process described matched all of the following criteria: (1) originated in the U.S., or was established by a U.S. agency or U.S.-based consensus organization; (2) described a stepwise process for a comprehensive exposure assessment, as previously defined; and (3) was intended to apply across a wide range of exposure scenarios. Peer-reviewed sources (scientific articles and book chapters) were included for review if the process described matched all of the following criteria: (1) the authors identified that the framework was to be used in the U.S. or all authors were from the U.S. or were noted as having an affiliation with a U.S. agency, consensus organization or U.S.-based company; (2) was cited in other documents as an established approach to exposure assessment; and (3) included information on a stepwise approach to a comprehensive exposure assessment, as previously defined.

Frameworks were excluded if they were designed solely for a specific substance, hazard, or exposure setting, or if they were not intended to be generalized beyond a single topic or scenario. For example, topic-specific models related to consumer product exposure or environmental fate modeling were identified but excluded from this review. Additionally, frameworks detailed for use around specific models or statistical approaches to data analysis were excluded. For example, some peer-reviewed sources specified the use of Bayesian analysis or high-throughput models and were thus excluded from this evaluation. Conceptual models for exposure assessment were also excluded, although they may also be referred to as frameworks by some organizations and authors.

In addition to the frameworks identified for the comparative analysis, some references were determined to have specific aspects that warranted exclusion from the overall analysis but were identified as foundational documents by the authors that helped inform the exposure assessment process, such as resource materials that were designed to enhance the exposure assessment discipline, or documents providing tools that could be used for a subset of the exposure assessment process. Although these frameworks are not included in the comparative analysis, they are addressed briefly in the Discussion.

Following the identification and initial review of the frameworks that met the inclusion criteria, each framework was compared step by step. Overlapping elements were identified, terminological inconsistencies were noted and aligned, and gaps were evaluated. Based on this comparative analysis, a consolidated framework was developed to integrate the essential elements of all reviewed sources. The resulting UEAF was organized into a stepwise format and example questions to be considered for each step were provided for practitioners. Further, it was identified that there could be potential deviations from the specific stepwise process presented in the UEAF and some of those nuances are also addressed briefly in the Discussion.

## Results

From review of key U.S. regulatory bodies, consensus organizations, and peer-reviewed scientific articles and book chapters, 99 exposure assessment approaches (including books, reports, standards, technical manuals, and webpages) were reviewed (Refer to Supplementary Reference list for full list of documents reviewed). Across the reviewed frameworks, analysis of the sequence and structure of recommended steps indicated variability in how the exposure assessment process is initiated. Some frameworks began with defining the study population, others with data collection, and others with broader framing of the assessment objective prior to population characterization or information gathering. This comparison highlighted problem formulation as a foundational step that shapes the scope and direction of the assessment. The stated objective of an exposure assessment influences the types of information to be collected, including both population characterization and data collection strategies, and whether the assessment is conducted retrospectively using existing information or prospectively through new data generation. This observation is consistent with broader risk assessment guidance emphasizing the importance of early consideration of decision context (2).

Evaluation of the frameworks also indicated variability in how final steps of the exposure assessment process are defined. While several frameworks concluded with analysis of uncertainty, others explicitly included communication of results to stakeholders and documentation of assessment methods and findings. Communication and recordkeeping were consistently identified as elements supporting transparency and consistency across assessments. In addition, consideration of repeatability and periodic reassessment emerged as cross-cutting elements associated with maintaining reliability of exposure characterization over time. The importance of transparent and reproducible exposure assessment approaches is also reflected in epidemiological literature, where limitations in exposure characterization can influence study findings (12, 13).

Of the 99 exposure assessment approaches (including books, reports, standards, technical manuals, and webpages) reviewed, based on the inclusion criteria, five distinct stepwise processes (i.e., frameworks) were identified for inclusion in this comparative review. These specific frameworks provided the detailed steps needed to perform a comprehensive human health exposure assessment, beginning with identifying the purpose of the assessment and ending with communication of the results to stakeholders.

### Regulatory bodies

From a regulatory standpoint, the U.S. Environmental Protection Agency (EPA) was the only U.S. federal agency identified meeting the predefined inclusion criteria that published an exposure assessment framework to be included in this analysis. The EPA has two different published comprehensive frameworks: first, EPA’s *Guidelines for Human Exposure Assessment,* dated October 2019, which provides overarching guidance that is broadly applicable across environmental, residential, consumer, and occupational chemical exposure contexts, although the agency notes that the focus is on the non-occupational environment, and second, the *Draft Systematic Review Protocol Supporting TSCA Risk Evaluations for Chemical Substances Version 1.0: A Generic Systematic Review Protocol with Chemical-Specific Methodologies*, dated December 2021, which addresses chemicals to be evaluated under the purview of the Toxic Substances Control Act (TSCA) (3, 14).

EPA’s *Guidelines for Human Exposure Assessment* from 2019, originally published in 1992, is the Agency’s most rigorous guidance document for performing exposure assessments (3, 15). This guideline provides detailed procedures for planning, scoping, data collection, modeling, and communication of exposure assessment efforts. In it, EPA states, “exposure research iterates between methods, measurements and models to develop scientific understanding and principles” and described five overarching considerations for developing the general approach to an exposure assessment, including exposure assessment design, tiered approaches, population selection approaches, estimation approaches, and stressor evaluation [(3), p. 7]. The guideline also considers special subpopulations for which additional considerations may be needed in exposure assessment, including children, workers, and other potentially vulnerable or highly exposed groups. Among these, children represent the only subpopulation for which multiple agencies and authors have proposed broadly applicable, framework-level considerations that extend beyond chemical- or scenario-specific guidance. These considerations, along with other published documents addressing exposure assessments for children, are explored further in the Discussion.

The other identified framework developed by EPA, the *Draft Systematic Review Protocol Supporting TSCA Risk Evaluations for Chemical Substances Version 1.0: A Generic Systematic Review Protocol with Chemical-Specific Methodologies* from 2021, “applies systematic review principles in the development of its risk evaluations of existing chemicals designated to be of high-priority or requested by manufacturers under the amended Toxic Substances Control Act (TSCA)” and noted that one of the goals was to customize and adopt systematic review protocols to “more than just hazard data” [(14), p. 24, 144]. It also established a hierarchy of exposure assessment data or input quality for assessing both environmental and occupational exposures. Although the 2021 report by the EPA is marked as a draft, the Agency is currently (as of 2026) relying on it as a vital resource for its TSCA risk evaluation process.

### Consensus organizations

Within the OEHS community, public health consensus organizations have provided voluminous practical guidance for exposure assessment. However, the American Industrial Hygiene Association (AIHA) was identified as the only consensus guidance organization meeting the predefined inclusion criteria that has published comprehensive frameworks or processes specific to exposure assessment.

Specifically, AIHA has two different published frameworks that met the inclusion criteria for this review: first, the *Guideline On Occupational Exposure Reconstruction* from 2008 edited by Viet et al., and second, *A Strategy for Assessing and Managing Occupational Exposures* textbook, commonly referred to as the Exposure Assessment Strategies book, last updated in 2015 and edited by Jahn et al. (16, 17). These texts emphasize iterative, stepwise, cyclical strategies that integrate professional judgment, data collection, modeling approaches, statistical tools, and risk communication.

AIHA’s *Guideline on Occupational Exposure Reconstruction* was created through a consensus process for occupational exposure reconstructions. As mentioned previously, exposure reconstructions are performed retrospectively after data has already been collected or specific activities are no longer performed. Through this guideline, an OEHS practitioner can perform a retrospective exposure assessment to help inform an occupational epidemiology study, a medical or hazard surveillance assessment, a review of health complaints, or a risk assessment (17).

The AIHA book *A Strategy for Assessing and Managing Occupational Exposures* provides an overview of recommended steps to be followed for both retrospective and current or prospective exposure assessments. The overall strategy of the exposure assessment process is the focus of this book, but the individual elements are detailed in a stepwise fashion for ease of review (16). These steps generally include a basic characterization of the exposure scenario of interest; iterative completion of the exposure assessment process, including an analysis of the duration, frequency, and intensity of exposures; a determination (or judgment) of the acceptability of the exposure assessment results, including the need for controls, further assessment including additional exposure information gathering, or future reevaluation of the same scenario; and finally, a cyclical reassessment process for all scenarios using an appropriate schedule.

### Peer-reviewed scientific articles and book chapters

Based on an examination of the peer-reviewed literature, one additional publication met the inclusion criteria identified and has helped advance exposure assessment framework development, the peer-reviewed publication titled *The Role of Exposure Reconstruction in Occupational Human Health Risk Assessment: Current Methods and a Recommended Framework* prepared by Sahmel et al. from 2010 (18). Although many other peer-reviewed publications addressing various aspects of exposure assessment and exposure science were identified, none of these presented a stepwise framework that was specifically applicable to comprehensive exposure assessment. Nonetheless, some of these important references and their relevance to the exposure assessment process are examined further in the Discussion.

The Sahmel et al. framework focused on retrospective exposure assessment and reconstruction, an area of practice often marked by substantial uncertainty and limited data. Their approach emphasizes the identification of available historical information, critically evaluating its quality, and documenting assumptions used to bridge data gaps, highlighting the importance of a stepwise approach that incorporates consideration of data quality, variability, and uncertainty analyses. A central feature of their work is the explicit inclusion of variability and uncertainty analyses as a standalone step, ensuring that retrospective exposure assessments are not presented as absolute values but instead characterized by ranges and levels of confidence. This methodological clarity is particularly influential because retrospective assessments are often conducted in contexts of litigation, compensation, or epidemiological research where credibility and defensibility are paramount.

For the present analysis, the Sahmel et al. framework demonstrates how a structured, stepwise process can guide practitioners even when the available exposure information is incomplete or when exposure data have substantial uncertainties associated with them. Although focused on retrospective exposure assessment, the framework highlights that the principles underlying occupational exposure assessment, including problem formulation, data gathering, scenario characterization, and communication of variability and uncertainty, are applicable across both historical and prospective exposure assessment contexts.

### Development of an overarching alignment framework—the Unified Exposure Assessment Framework (UEAF)

By combining the regulatory, scientific, and professional concepts from the frameworks identified above, the UEAF was created to provide a comprehensive, practical, and broadly applicable framework across exposure domains. The UEAF integrates explicit steps for evaluating data quality, documenting assumptions, and considering uncertainty throughout the assessment process. In situations where historical exposures must be evaluated or reconstructed, the applicability of the UEAF is extended beyond compliance monitoring to research and forensic applications.

An analysis of each of the above approaches or frameworks indicated that the recommended steps often overlapped with one another, although the differences in terminology and the details specified under each step are apparent in each of the frameworks. The following table provides the steps of the UEAF and provides a visual pathway for which steps were identified within each framework (Table 1). The final list of steps and considerations, as cross-walked across all identified sources, ultimately forms the basis for the UEAF. Each step of the UEAF is outlined below, identifying their needs and considerations. The comparison to the five identified frameworks are further considered and expanded below in the Discussion. Overall, the UEAF is designed to consolidate the published frameworks and create a single stepwise process that includes the nuances of each of the published frameworks for ease of access for OEHS and exposure science practitioners to ensure that an exposure assessment can be conducted with all of the elements of the identified comprehensive U.S. exposure assessment frameworks available in the literature.

**Table 1:** Unified Exposure Assessment Framework (UEAF) steps with condensed overview of identified, published exposure assessment frameworks in the U.S.

UEAF steps^*^	AIHA/Viet et al. (17)	Sahmel et al. (18)	AIHA/Jahn et al. (16)	EPA (3)	EPA (14)
Goal setting and scoping	✓	✓	✓	✓	✓
Scenario characterization	✓	✓	✓	✓	✓
Establish exposure pathways/similar exposure populations (SEPs)			✓		
Data collection	✓	✓	✓	✓	✓
Characterization of variability		✓	✓	✓	✓
Analysis of uncertainty	✓	✓	✓		
Validation		✓			
Reporting, communication and recordkeeping	✓	✓	✓	✓	✓

*Reassessment/Review was specified as a step for some identified frameworks, but it was determined that this should be performed at every step to ensure proper review of data.

UEAF Step 1—Goal Setting and Scoping: The initial step of the UEAF defines the objectives and boundaries of the exposure assessment. Considerations for this step include identifying the problem that exists, designing the exposure assessment, determining feasibility and identifying any data available for the exposure assessment prior to any forward progression.

UEAF Step 2—Scenario Characterization: Characterizing the scenario under Step 2 of the UEAF establishes a structured understanding of the exposure setting before engaging in the full exposure assessment process. This step considers the population of individuals who are part of the scenario and what tasks and agents are involved in the exposure scenario.

UEAF Step 3—Establish Exposure Pathways/Similar Exposure Populations (SEPs): Step 3 of the UEAF focuses on determining the pathways (i.e., routes) of exposure being characterized for the exposure assessment (e.g., dermal, oral, inhalation), and grouping the population of individuals based on their similarities in exposure, which includes the agents to which they are exposed, the pathway of exposure and the tasks performed that may result in said exposure, including the duration and frequency of such exposure.

UEAF Step 4—Data Collection: Step 4 of the UEAF brings together Steps 1 through 3 by determining if data needs to be collected based on the needs of the assessment, and if data are needed, then it establishes the type of data to collect for the exposure assessment, whether it will be quantitative, semi-quantitative, or qualitative, the types of samples to be collected and if modeling may be needed in addition to, or in lieu of, collecting samples.

UEAF Step 5—Characterization of Variability: Variability within an exposure assessment can be found in differences temporally, spatially and between and within the population being studied. These differences can lead to underestimation or overestimation of risk for a certain population. Therefore, UEAF Step 5 ensures that the inherent variability of a dataset is identified and considered rather than overlooked or ignored.

UEAF Step 6—Analysis of Uncertainty: Uncertainty within an exposure assessment is based on gaps in knowledge within the assessment, which can be due to lack of data, errors in certain parameters, or the assumptions used for certain variables or within modeling. Through analyzing uncertainty in UEAF Step 6, reducing and characterizing uncertainty in the process is possible.

UEAF Step 7—Validation: The validation step under UEAF Step 7 involves comparison of the exposure assessment outcome to a second line of evidence or secondary source of information. Through this step, repeatability is also considered as both a quality assurance measure and a mechanism for continuous improvement by multiple practitioners.

UEAF Step 8—Reporting, Communication and Recordkeeping: Reporting and communication of the results of the exposure assessment ensures that those relying on the assessment understand its purpose, scope, limitations, and conclusions. The audience must be considered; different stakeholders may find various aspects of the exposure assessment more or less important. Recordkeeping allows exposure assessments to serve as living documents that can be revisited and updated as processes, standards, or environmental conditions evolve.

Figure 1 presents the UEAF steps with their primary objectives. In addition to the outlined steps, targeted example questions to assist practitioners in framing out the needs for each step presented in the UEAF are provided in Figure 2. The example questions in Figure 2 are intended as illustrative sample prompts, through which practitioners can begin discussions for each step for any exposure scenario.

**Figure 1:**
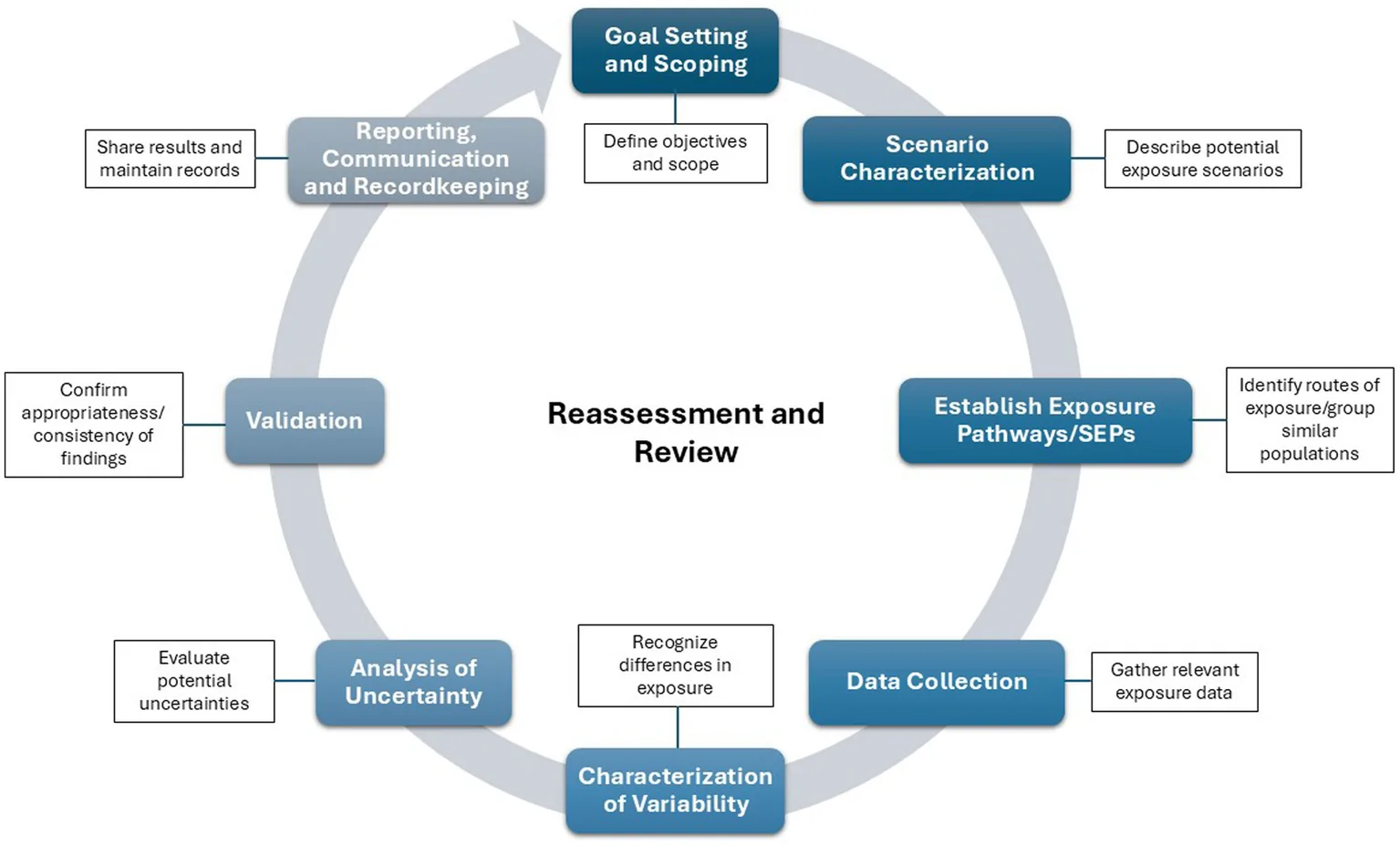
Unified Exposure Assessment Framework steps.

**Figure 2:**
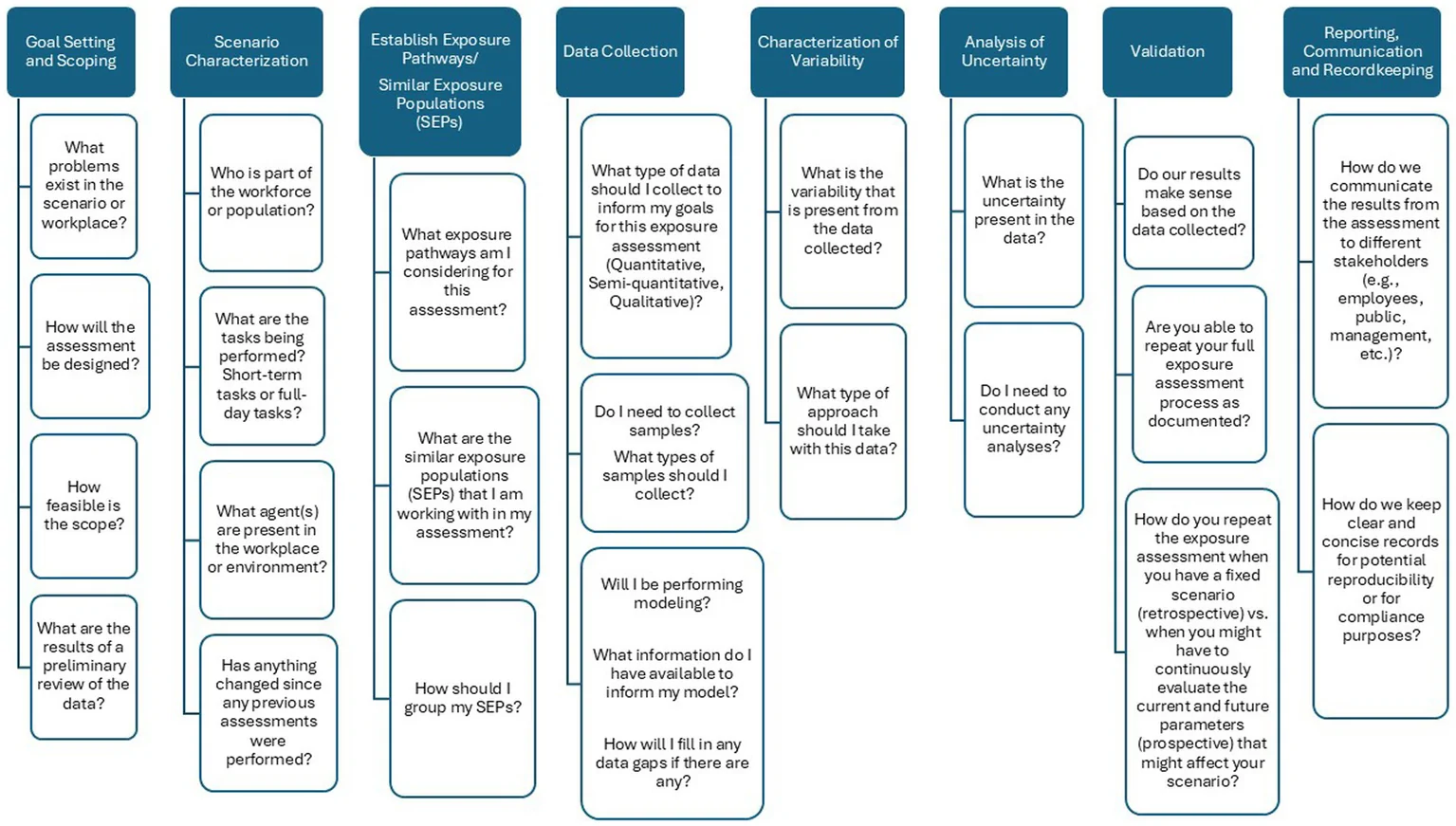
Questions to consider for each step of the UEAF.

## Discussion

### Alignment of UEAF steps across frameworks

Each step of the UEAF was created from the existing identified occupational and environmental human health exposure assessment frameworks in order to interpret and highlight areas of alignment and divergence. As visualized in Table 1, the comparison across existing frameworks indicates that the harmonized UEAF steps accurately represent the intent and structure of the underlying frameworks while preserving their distinct emphases. The UEAF helps to strengthen consistency and applicability across exposure assessment practices in both occupational and environmental settings.

#### UEAF step 1—goal setting and scoping

UEAF Step 1 aligns closely with the problem formulation phases outlined in EPA as well as the goal establishment step outlined by Sahmel et al. and Viet et al., where the exposure assessment’s purpose and limits of the process ensures that subsequent analyses are contextually appropriate and decision-relevant (3, 17, 18). Across occupational and environmental approaches, this emphasis on early assessment foundation establishes that explicit goals drive data efficiency and interpretive transparency, as well as relevancy of results. The Exposure Assessment Strategies book from AIHA integrates comparable practices into professional guidance to maintain internal consistency and reproducibility (16).

From a comparative standpoint, this step illustrates a strong convergence across frameworks despite differences in scope and terminology (for example, environmental vs. occupational exposures). Perhaps not surprisingly, occupational exposure assessment guidance tends to frame goal setting around worker populations and specific occupational tasks, whereas environmental exposure assessment frameworks emphasize receptor populations and exposure pathways. Interpreting these in parallel shows that the UEAF approach provides a shared structure adaptable to both contexts without altering foundational intent. This alignment supports consistent application of exposure assessment principles, enabling cross-domain comparability and reinforcing the scientific defensibility of early-stage planning decisions.

Some of the frameworks also stress the importance of engaging a qualified professional, or a qualified industrial hygienist, to conduct the assessment (3, 16). Although not in every identified approach, proper qualifications are an important part of the process to ensure data are reviewed accurately and appropriate steps are taken towards making decisions needed for the assessment. Individuals with credentials such as the Board for Global EHS Credentialing (BGC) Certified Industrial Hygienist (CIH) and/or the AIHA Registry Programs Registered Specialist in Exposure Decision Analysis (EDA) can bring a solid foundation to the underlying needs of the exposure assessment process. Additionally, all exposure assessments may require a team of individuals to provide input. As recommended by the EPA, individuals with expertise in exposure assessment, human health, risk assessment and risk management should all be considered for inclusion on the exposure assessment team as they can provide specific information that will be necessary for a comprehensive assessment, depending on the goals and scope (3). The EPA also specifically recommends that the team should incorporate, or consult with, professionals with expertise in quantitative exposure modeling and statistics where relevant.

Reassessment and review during this step involve identifying the problems that need to be solved through the exposure assessment process. As questions arise, the practitioner must determine if additional questions should be answered through the process, or if the assessment should be limited. Additionally, this may be an iterative process in which the scope is refined based on the feasibility of the assessment according to data availability and access, economic or cost considerations, level of detail required for the assessment results, and other resource considerations.

#### UEAF step 2—scenario characterization

For occupational scenario characterization, AIHA emphasizes defining relevant exposure groups, determinants, and activities to ensure representative characterization of each exposure (16). In parallel, for non-occupational settings or general exposure scenarios, EPA describes scenario characterization as the organization of contextual information, sources, pathways, environmental media, and exposed populations necessary to support conceptual model development (3). Although not identified as a separate step by Sahmel et al. and Viet et al., the importance of the scenario characterization concept is present through their discussions on review of the available information needed for the exposure reconstruction (17, 18). These approaches highlight the value of assumptions and boundary conditions, reinforcing that an accurate exposure scenario definition is essential for both risk interpretation and communication.

A key point of convergence across frameworks is the treatment of data sources within this step. EPA uses an exposure assessment information hierarchy that prioritizes the use of scenario-specific empirical measurements where available, followed by surrogate data for other hazards for similar scenarios or other scenarios for similar hazards, followed by modeled estimates, and finally default threshold-based approaches (such as use of existing exposure limits), qualitative approaches or expert judgement, with each tier of information evaluated for quality and relevance (3, 14). Although potentially important depending on the scenario and the overall availability of information, qualitative descriptions and professional judgement do not provide as much precision and accuracy compared to quantitative exposure data and semiquantitative modeling results and may be inappropriate as a sole baseline for the exposure assessment (18). This principle parallels the data evaluation and professional judgment approaches found in the AIHA framework, where selection of information is guided by intended use and uncertainty tolerance. By incorporating this hierarchical concept, the UEAF promotes consistency in how occupational and environmental scenarios are defined and documented. This integration supports accurate characterization across diverse contexts and reinforces the framework’s emphasis on transparency, comparability, and iterative refinement, depending on the goals and needs of the specific assessment.

Reassessment and review occurs during this step when a practitioner has collected all the information they have available and determines if that data answers any of the questions posed in Step 1, or if additional issues or gaps are brought to their attention that must be answered before the goals of the assessment are met. This approach also forms the basis for a tiered assessment.

#### UEAF step 3—establish exposure pathways/similar exposure populations (SEPs)

Step 3, establishing exposure pathways and similar exposure populations, reflects broad agreement among occupational and environmental frameworks on the need to link sources, pathways, and receptors. In occupational practice, AIHA emphasizes grouping workers into SEGs to characterize exposure potential for single types of job-based exposures or tasks to single hazards, while EPA applies parallel logic to environmental receptors and exposure pathways, in addition to examining aggregate or cumulative exposures where appropriate (3, 16). To align the use of the UEAF with non-occupational and general population exposure settings, this review uses the term Similar Exposure Populations (SEPs). While EPA and NRC do not use a term such as SEP specifically, it was developed for the purposes of this framework as an extension of the occupational SEG concept to general population and non-occupational settings. This terminology is consistent with broader exposure science concepts described by NRC, including receptor populations, source-to-receptor and source-to-dose pathways, population-level exposure variability, vulnerable or hot-spot communities, and exposure determinants such as route, medium, geography, activity patterns, and other behavioral or demographic factors (2, 7). Each approach relies on articulation of the factors that drive contact and variability, such as activity patterns, environmental media, and time–location relationships, to ensure that exposure estimates are both relevant and transparent. Again, although not explicitly identified as a separate step in Sahmel et al. and Viet et al., the importance of the establishing exposure pathways and SEPs is present through their discussions on the review of the available information needed for the exposure reconstruction (17, 18).

This step also represents the point at which planning both the current assessment and any future exposure assessments begins in earnest. By using available information to characterize present exposure conditions, practitioners can evaluate data adequacy, identify critical data gaps, and determine whether existing measurements or models are sufficient to meet the assessment objectives. This approach also informs decisions about whether additional monitoring, data collection, or refined modeling may be required in future iterations or tiers of the assessment to improve confidence or support decision-making. SEPs may also need to be defined differently for different exposure pathways or routes; for example, the SEP for a dermal exposure profile may look quite different than the SEP for an inhalation exposure profile depending on the scenario.

Reassessment and review occur in an assessment as SEPs or receptor groups are refined; data evaluation may reveal that certain populations differ more than initially expected, prompting subdivision into multiple SEPs, or that process or task changes warrant combining groups into a larger, single SEP. This iterative refinement, emphasized across both AIHA and EPA guidance, illustrates how the UEAF incorporates flexibility without compromising structure. By encouraging continuous review of groupings and pathways, the framework maintains scientific clarity and adaptability across occupational and environmental contexts.

#### UEAF step 4—data collection

Across frameworks, data collection is routinely presented as a structured and iterative process rather than a single event. This step marks the transition from conceptualization to assessment. As discussed previously, EPA and Sahmel et al. outline a tiered data hierarchy that prioritizes direct measurements, followed by modeled estimates, then when necessary and more robust data are not available, default or surrogate data or qualitative information, each evaluated for quality and relevance, followed by exposure determinants identified and professional judgment detailed through an evaluation of available information, including quantitative and qualitative data (3, 18). Occupational approaches as noted by AIHA echo this principle by emphasizing the selection of data proportional to the assessment’s scope and uncertainty tolerance. AIHA also provides guidance for when and how to use modeling when monitoring data are unavailable or insufficient (16). Regarding data collection for exposure reconstructions, Viet et al. identifies that it should be transparent and protocols should be established (17). Collectively, these frameworks converge on the importance of documenting the basis for data selection and ensuring traceability of all inputs used to inform exposure estimates. They also ensure that the most robust data and information available, which also meet the goals of the assessment, are used since the type of data collected and used ultimately determines the quality of the assessment.

Reassessment often occurs as new data or modeling information are acquired, particularly when preliminary results challenge earlier assumptions about exposure pathways or population groupings. Frameworks such as EPA and AIHA explicitly promote this iterative refinement to improve precision while maintaining efficiency (3, 16). Within the UEAF, this approach supports adaptability across occupational and environmental contexts by encouraging assessors to use the highest-quality data available, revisit earlier judgments when warranted, and communicate data limitations that may influence interpretation.

#### UEAF step 5—characterization of variability

Recognizing and characterizing variability is treated as a central component of exposure assessment across all major frameworks. EPA describes variability as the natural heterogeneity in exposure determinants, such as behavior, environmental conditions, and emission dynamics, that should be captured, rather than averaged out (3). Occupational guidance from AIHA emphasizes evaluating how job tasks, control measures, and temporal factors contribute to within- and between-group variation, using that insight to determine the representativeness of collected data (16). Although not explicitly identified as a separate step in the frameworks by Sahmel et al. and Viet et al., the concept of variability characterization is discussed in processes specific to differences that can occur within-workers and between-workers (17, 18). Together, these perspectives establish variability assessment as a scientific exercise in describing the range and distribution of exposures rather than seeking a single point estimate.

Across both occupational and environmental settings, identifying variability also supports prioritization and adaptive planning. Patterns observed during this stage often prompt reassessment of exposure groupings, sampling frequency, or modeling resolution using a tiered approach. EPA encourages iterative evaluation to determine whether observed differences are intrinsic to the system or a result of data limitations. AIHA provides detailed statistical guidance on the expected standard deviation in the data that is likely to be appropriate for a single SEP to be considered truly similarly exposed. AIHA guidance identifies that data sets in occupational settings can typically be assumed to be lognormally distributed, which shows as a right-skewed distribution of data points, and the geometric mean and geometric standard deviation can be used to determine central tendency and measures of dispersion across the data set (16). Generally, in the AIHA strategy, as the geometric standard deviation increases, variability increases, so the sample size required in a sampling strategy also increases to maintain the same level of confidence in decision statistics, such as some upper tolerance limit of a percentile value, although typically for occupational data sets approximately 6–10 measurements are needed to achieve sufficient confidence depending on the metric used (16). EPA does not specifically provide a distribution fit that is typical for environmental settings in the framework cited in this review, and therefore the exposure assessor must determine the distribution type that is the most appropriate fit for the exposure data (e.g., normal, uniform, lognormal) (3). Statistical tools such as Monte Carlo analysis, Bayesian Decision Analysis and mixed effects models can be used to assess variability (3, 16, 19, 20). Integrating these concepts, the UEAF treats variability as a separate step that links scenario characterization, data collection, and uncertainty evaluation. This alignment fosters transparency in interpreting exposure distributions and supports the scientific coherence of assessments conducted across occupational and environmental domains.

Most of the frameworks evaluated either combined UEAF Step 5 and UEAF Step 6 (characterization of variability and analysis of uncertainty) into one step, or described variability characterization in other parts of the exposure assessment process without explicitly identifying it as its own step (3, 14, 17). However, because of the importance of and distinction between variability and uncertainty elements of an exposure assessment, the UEAF has them split into two separate steps as they are different concepts to address as part of the assessment process.

#### UEAF step 6—analysis of uncertainty

All major frameworks recognize that uncertainty analysis is inseparable from interpreting exposure results. Both EPA frameworks and Sahmel et al. describe uncertainty analysis as a systematic evaluation of incomplete or imperfect knowledge, distinct from variability (3, 14, 18). These frameworks recommend explicitly identifying key sources of uncertainty, such as data gaps, measurement limitations, model assumptions, and exposure factor estimates, to provide context for confidence in conclusions. Occupational guidance from AIHA parallels this approach by embedding uncertainty considerations within professional judgment and emphasizing clear documentation of methods and assumptions (16). Some uncertainty exists in every exposure assessment. Collectively, these frameworks provide methods for successfully communicating uncertainties and their potential effects on the result.

Although terminology and analytical depth differ, the treatment of uncertainty is conceptually consistent across domains. As detailed by Sahmel et al., there are four levels of uncertainty analyses that can be applied to an assessment depending on the level of detail necessary in the assessment itself (18). These tiers include: (1) a screening-level uncertainty analysis using generic or default values published by regulatory or consensus organizations, (2) a qualitative identification of the types and individual sources of uncertainty in the assessment, (3) a semiquantitative or even quantitative deterministic sensitivity analysis to determine which parameters have the greatest effect on the uncertainty of the final results, and (4) utilization of probabilistic techniques to assess both the individual and combined effects from parameter uncertainty in the assessment (18). Viet et al. identifies that uncertainty assessments can be used as a quality assurance activity (17). Environmental frameworks often formalize uncertainty analysis using structured qualitative or quantitative approaches, whereas occupational frameworks emphasize iterative review and expert evaluation. Both perspectives aim to ensure that confidence in conclusions reflects the strength of underlying evidence. Within the UEAF, these philosophies are combined through having uncertainty evaluated throughout the assessment rather than confined to the end of the process. This ongoing consideration allows practitioners to update confidence bounds as new data emerge or as scenarios evolve, maintaining proportionality between available information and interpretive confidence.

Uncertainty analysis extends beyond the exposure estimation process itself. Limitations in toxicological data, variability or uncertainty in the derivation of OELs, and the conservatism embedded in regulatory reference values all contribute to the total uncertainty associated with exposure decision-making. Incorporating this recognition, the UEAF positions uncertainty analysis as a cross-cutting activity that links exposure characterization with risk interpretation, promoting balanced, scientifically sound conclusions across occupational and environmental contexts.

Uncertainty analyses are important in understanding the most influential parameters in any assessment. Reassessment and review enters this step when determining the level of uncertainty that is acceptable to the practitioner in the assessment. If uncertainty is low, then the assessor may be comfortable with moving on to the next step, but if uncertainty in the data is high, it might be necessary to collect more data, or to determine if there was a gap in a previous step and perform a reevaluation and correction.

#### UEAF step 7—validation

The next step, validation, is addressed consistently across occupational and environmental frameworks as a means of confirming that exposure assessment results are representative, repeatable, and appropriate for their intended use. In the identified frameworks, the validation step may be defined using terms such as quality check/quality assurance, corroboration, or even reassessment. The EPA emphasizes the importance of evaluating data integrity, model performance, and consistency between predicted and observed results (3). Occupational frameworks such as AIHA echo these principles by recommending independent review or comparison against a second source such as established benchmarks, professional experience, or external datasets (16). AIHA specifically identifies multiple ways to validate an exposure assessment’s conclusions, including evaluation of a subset of the data with Bayesian Decision Analysis, or performing a full personal monitoring study to collect a statistically significant number of samples to determine that the assessment of exposures was appropriate, as well as laboratory studies that simulate real world exposure concentrations and modeling (16). Viet et al. identifies validation as a quality assurance activity that should be considered and promotes a number of options on how this can be completed using a secondary source of information, including the use of expert panels to evaluate quantitative results, comparison to a reserved set of data, use of simulated exposure studies, use of biomonitoring data if available and indirect validation using the findings of the study to confirm their information was correct (17). Sahmel et al. also identifies validation as an important step in the exposure reconstruction process using similar types of approaches, and notes that it can help increase the confidence in the results from the process (18).

The source documents for the comprehensive frameworks recognize the importance of documentation, trackability, and consistent procedures in exposure assessment. EPA emphasizes that detailed documentation and traceable data workflows enable assessments to be revisited or replicated when new information arises (3). Occupational guidance from AIHA highlights the importance of maintaining consistent procedures for data collection, interpretation, and recordkeeping (16). These two examples demonstrate the importance of reproducible assessments for credibility and consistency. Scientifically sound exposure assessments should be able to be generally repeated by a separate assessor by providing a logical and traceable set of inputs, methods and results, along with a sound, documented conclusion (or result). This supports consistency, facilitates future reviews or assessment, and helps practitioners document exposure assessments in a way that can be followed over time.

Reassessment during this step often occurs when new information, such as updated process data, revised exposure limits, or additional measurements, challenges previous findings or conclusions. Frameworks from EPA and AIHA both recognize that this process supports reproducibility and fosters continuous improvement rather than serving as a one-time verification. Within the UEAF, validation operates as a feedback mechanism that links uncertainty analysis, data collection, and reporting. By integrating validation throughout the assessment process, the framework allows the results to remain credible, relevant, and adaptable to changing occupational and environmental conditions. This step largely focuses on reviewing the conclusions that have resulted from the exposure assessment and determining if these conclusions make sense by using secondary lines of evidence, and allows the practitioner to determine if additional information is needed to ensure the assessment is repeatable. The reassessment and review step is integrated within this step and is therefore not standalone.

#### UEAF step 8—reporting, communication and recordkeeping

Communication and documentation practices are central features of every major exposure assessment framework. EPA treats communication as an integral part of the scientific process, emphasizing the need for transparency, reproducibility, and accessibility of methods and results (3). The occupational framework from AIHA places similar emphasis on clarity, recommending that findings be presented in a form that is actionable and tailored to different audiences, including workers, management, regulators, and other stakeholders (16). Across frameworks, communication is not an endpoint, but an ongoing component of the assessment process.

Reporting conventions also show conceptual alignment across domains. EPA emphasizes structured reporting formats that trace the logic from scoping through data interpretation, allowing future reviewers to reconstruct decisions (3). Similarly, AIHA advocates for written reports that clearly describe exposure groups, sampling strategies, analytical methods, and interpretation of results (16). These frameworks indicate that effective reporting is important for supporting the utility and credibility of exposure assessments. Although the communication style of the results, or of the entire process, may differ depending on the audience and goals of the assessment, the assessment should include communication of relevant methods, assumptions, limitations, and conclusions, whether for compliance, organizational decision-making, or, at a minimum, transparency purposes.

Recordkeeping and data retention are equally emphasized. EPA guidance stresses maintaining detailed records to support future reassessment, model validation, and quality assurance, which is mirrored by AIHA, which notes that every element of the assessment should be documented and the practitioner should consider application of the assessment results by future practitioners and how their data would be reviewed and interpreted (3, 16).

Finally, the UEAF integrates these communication and recordkeeping elements into a unified structure applicable across occupational and environmental contexts. The framework includes reporting of data sources, assumptions, and uncertainty alongside explanations of how results will be used in decision-making. By embedding communication and documentation from the beginning of the assessment rather than confining them to its conclusion, the UEAF emphasizes collaboration among technical experts, management, and affected communities where appropriate. This continuous exchange of information can improve clarity, support stakeholder understanding, and allow exposure assessments to be revisited or updated over time.

#### Reassessment and review

Reassessment and review are intentionally embedded throughout the UEAF rather than represented as a discrete step in the process. Reassessment is encouraged whenever new data, technological advances, or changes in work or environmental conditions alter exposure potential. This iterative approach mirrors the review loops described in EPA guidance, where each reassessment refines assumptions, strengthens data interpretation, and improves decision-making confidence. As illustrated in Figure 1, this process operates cyclically, encouraging periodic evaluation of goals, data quality, exposure groupings, and assumptions as new information becomes available. EPA identifies an iterative review as essential for maintaining scientific integrity, while AIHA emphasizes that exposure assessment decisions must be revisited when workplace or environmental conditions change (3, 16). This recurring theme appears in multiple steps of the UEAF, from initial scoping and scenario characterization through data collection, variability analysis, and validation, demonstrating that continuous review sustains transparency, adaptability, and long-term reliability across occupational and environmental exposure assessments.

#### UEAF overview

Overall, the proposed UEAF identifies each step needed to perform a comprehensive exposure assessment by combining essential elements of the identified published U.S. exposure assessment frameworks and further captures nuances within each source framework. This framework provides a unified, organized sequence that helps strengthen consistency and applicability across exposure assessment practices in both occupational and environmental contexts and allows OEHS and exposure science practitioners to perform a repeatable, comprehensive exposure assessment.

### Additional resources and tools for comprehensive exposure assessments

#### Foundational resources from the NAS

The NRC’s Red Book identified exposure assessment as an essential step in the risk assessment process and was subsequently expanded upon by the more recent NRC “Silver Book” of 2009, which refined the original risk assessment paradigm while retaining foundational concepts introduced in the Red Book (1, 2). These reports have informed many existing U.S. exposure assessment frameworks despite their broader focus on risk assessment.

The National Academies’ risk assessment publications provide important conceptual foundations for the UEAF by emphasizing problem formulation, systematic evaluation of evidence, and communication of assumptions, variability, and uncertainty (1, 2). The NRC’s 2009 report further highlighted the importance of defining decision context, scope, and information needs prior to data collection or analysis, reinforcing the role of goal setting and scoping as an initiating step for exposure assessment. These principles have informed regulatory and professional guidance and provide a conceptual basis for structuring exposure assessments as transparent, decision-relevant processes.

Subsequent NAS/NRC publications expanded the scope of exposure science by emphasizing integration across pathways and time scales, incorporation of emerging tools and data streams, and the need for adaptable, organized approaches to exposure characterization (5, 7). Collectively, these reports highlight the importance of transparency in data origin and quality, consideration of uncertainty, and clear documentation to support interpretation and future reassessment, reinforcing the value of a structured framework that can accommodate both established and evolving exposure assessment methods.

#### Resources that enhance the exposure assessment process

Multiple frameworks and tools fall in the category of resources that have enhanced the exposure assessment process. The amount of literature on this topic is vast, so notably the resources identified here only represent a small subset of those available that can be used to bolster an exposure assessment.

The AIHA *Principles of Good Practice* (PGP) technical framework from 2024 was identified as a critical resource for OEHS and exposure science practitioners. Specifically, the AIHA PGP was created as a collection of guidelines that are used by OEHS practitioners around the world for protecting their workers and communities from unacceptable risks. It was noted that the PGP was “neither comprehensive nor complete” and should be followed as a “foundational program and practice needs” guidance document [(21), p. 5]. The PGP relies on the AIHA’s more comprehensive framework and each OEHS process/program identified provided practices that were considered to be “good” or “enhanced” based on the collected data of practitioners around the world, which makes this an important resource for practitioners.

Several widely used publications and guidance documents address specific components of exposure assessment but do not describe a comprehensive, end-to-end stepwise process and therefore were not included in the comparative framework analysis. Because these resources remain influential in exposure science and practice, they are briefly described here for context. These resources primarily provide technical methods, tools, or programmatic guidance. Neither the Occupational Safety and Health Administration (OSHA) nor the National Institute of Occupational Safety and Health (NIOSH) have an established exposure assessment framework. Although OSHA was the first identified regulatory agency requiring occupational exposure assessment, no stepwise exposure assessment framework developed by OSHA was identified for review, although there is an OSHA stepwise framework for hazard identification and assessment (22). Additionally, although NIOSH has the Current Intelligence Bulletin 69 titled *NIOSH Practices in Occupational Risk Assessment*, they explicitly state that they do not follow the exposure assessment step found in the traditional NRC risk assessment process for their own risk assessments and “[i]n contrast, NIOSH uses the information from the first two steps of the risk assessment to estimate health risks of individuals from the agent at various levels of exposure” [(23), p. vii]. However, both OSHA and NIOSH provide manuals that detail tools practitioners may need for certain elements of an exposure assessment, such as what can be used in the UEAF data collection step. For example, the OSHA Technical Manual includes a chapter on sampling strategy and guidelines, which provides practitioners with useful information on how to sample air contaminants (24). Likewise, the NIOSH Manual of Analytical Methods provides practitioners with analytical methods useful for workplace monitoring (25). NIOSH has also published the Occupational Exposure Sampling Strategy Manual, which provides early technical guidance on exposure monitoring program design and statistical sampling approaches (26). In addition to these tools which practitioners can use, the EPA has also developed the Integrated Risk Information System to provide a database on “human health effects that may result from exposure to various chemicals in the environment,” which can be utilized for exposure assessment information (27).

In addition to the resources detailed from OSHA, NIOSH and AIHA, the American Conference of Governmental Industrial Hygienists (ACGIH) also provides guidance on exposure assessment for OEHS and exposure science practitioners in the *Modern Industrial Hygiene* book from 2008 (28). This book describes methods for exposure evaluation but focuses on specific steps or elements within the defined exposure assessment process and thus was not included in the comparative analysis. The ACGIH book serves as a reference that integrates foundational industrial hygiene principles with applied methodologies for exposure evaluation and control on specific topics. Within this text, chapters 12, 13, and 14, provide a structured, iterative methodology that forms the basis for modern exposure assessment practice and aligns conceptually with the tiered, data-driven approaches, although presented in a less detailed manner than the frameworks included in this analysis.

Another review of exposure assessment methods is presented in a book chapter authored by Paustenbach et al. in 2024. In this chapter, which is a more recent version to a previously published work discussing the state of the science for exposure assessment by Paustenbach et al. from 2000, the authors offer a review of exposure assessment practices, synthesizing decades of experience and scientific literature into a modernized set of methods for practitioners (29, 30). Their review underscores the iterative and cyclical nature of exposure assessment, stressing the role of professional experience in shaping study design, data collection, and interpretation. The authors emphasized the integration of quantitative tools such as statistical analyses and exposure modeling, along with qualitative methods such as scenario-based reasoning and expert elicitation. Importantly, the review highlighted that exposure assessments must be both systematic and transparent in order to be credible, reproducible, and broadly applicable across exposure settings.

One work that focused on a discrete element of the exposure assessment process, which provides detailed methodological guidance that complements broader frameworks, is the Aggregate Exposure Pathway (AEP) construct analysis by Teeguarden et al. from 2016 (31). The authors presented the Aggregate Exposure Pathway (AEP) construct, which is useful for organizing information on exposure sources, pathways, and internal dose, but did not address core framework elements such as problem formulation, study design, communication, or reassessment. Other component-focused evaluations have emphasized analytical tools used within exposure assessments rather than the overall assessment process itself. Jayjock et al. provided a detailed review of an exposure modeling framework to be used in occupational hygiene assessments, highlighting model selection, parameterization, and appropriate interpretation when empirical data are limited (32). Sexton et al. focused on biomonitoring and tissue measurements, discussing their value for integrating exposures across pathways while also noting limitations related to interpretation and temporal resolution (33). Additionally, Seixas and Checkoway presented a rigorous framework for retrospective exposure assessment in occupational epidemiology, emphasizing historical exposure reconstruction, evaluation of exposure misclassification, and explicit consideration of uncertainty when developing exposure metrics for health outcome studies (34). While these contributions are important to exposure science, they addressed specific methods applied within an exposure assessment rather than defining a structured sequence of steps guiding an assessment from planning through communication and reassessment.

Similarly, the Agency for Toxic Substances and Disease Registry (ATSDR) maintains an electronic manual entitled Public Health Assessment Guidance Manual (PHAGM), which provides structured guidance for site-specific public health evaluations. The manual provides steps to follow specific to hazardous waste sites and environmental health emergencies, with a strong emphasis on pathway identification and screening-level decision making (35).

Notably, the EPA had a wide breadth of guidance for exposure assessment in its foundational *Guidelines for Exposure Assessment* published in 1992, as well as additional frameworks such as the *Framework for Human Health Risk Assessment to Inform Decision Making* from 2014, to more scenario-specific guidance for superfund sites or pesticide exposure (15, 36–38). These publications from the EPA were reviewed but not included in the comparative analysis due to their scenario-specific nature, and because the more recent EPA 2019 document was seen as the update to the earlier documents and in many instances was identified as the framework applied in these more specific types of assessments (whether the 1992 or 2019 edition).

Collectively, these component- and application-specific frameworks underscore the depth and maturity of exposure science methods while reinforcing the need for an overarching, integrative framework to guide practitioners in selecting, sequencing, and applying these methods within a transparent, purpose-driven exposure assessment process.

#### Population-specific assessment considerations (specifically children)

Population-specific exposure assessment considerations, particularly those related to children, represent an important category of modification to the generalized UEAF steps. Numerous studies and guidance documents describe frameworks or framework elements tailored to children that emphasize differences in exposure pathways, activity patterns, and physiological characteristics compared to adults (39–46). These publications consistently highlight the importance of incorporating age-specific exposure determinants, toxicokinetic considerations, and pathway-specific behaviors when characterizing exposures in children. Collectively, this body of work demonstrates that while the overall structure of exposure assessment remains applicable, modifications may be needed within specific UEAF steps, particularly during scenario characterization, exposure pathway identification, and data collection.

Research evaluating children’s exposure determinants indicates that behavioral and physiological differences, such as higher intake rates relative to body weight, distinct activity patterns, and developmental considerations, can influence exposure magnitude and variability (43, 45, 47). Practical challenges associated with monitoring children, including methodological limitations and ethical considerations, may also increase uncertainty and influence data collection approaches (42). These factors reinforce that population-specific characteristics should be considered when applying the UEAF to sensitive or highly variable populations, particularly during scoping, scenario characterization, data collection, and uncertainty evaluation.

#### Exposure and control banding

In the U.S., exposure banding has been developed as a supporting approach within occupational risk assessment to provide health-based concentration ranges when authoritative occupational exposure limits are unavailable. In contrast, control banding integrates hazard and exposure potential to recommend control strategies and is therefore considered a risk management activity rather than an exposure assessment activity (48–50).

Exposure banding represents a supporting analytical tool that may be applied within framework steps related to interpretation of exposure estimates, uncertainty evaluation, or decision context. Related categorization-based approaches have also been applied within pharmaceutical and broader hazard banding contexts (51–53) with more recent extensions to computational applications for data-poor substances (54).

### Potential modifications of the UEAF

Although the UEAF structure is intended to apply broadly across exposure scenarios, modifications may be warranted for various types of exposure assessments. With focus on the UEAF, practitioners might recognize a potential need for deviating from the stepwise process presented but still utilizing the framework as a whole. Two scenarios where this type of adjustment may occur are explained below.

#### Retrospective vs. prospective exposure assessments

There are some unique stepwise differences that become apparent in the approach to retrospective exposure assessments or exposure reconstructions when compared to prospective or current exposure assessments. In prospective exposure assessments, the process and approach can be scoped to specifically meet the goals of the assessment, whereas for retrospective scenarios, there can be constraints with respect to what data were previously collected or available and how they can be used in the evaluation (18). For example, the goal setting and scoping step of a retrospective exposure assessment may involve a feasibility consideration that is based on different factors compared to a prospective assessment, such as an evaluation of data availability rather than an analysis of the cost of data collection and analysis. Additionally, the data collection step may need to be performed before the exposure analysis steps in the process, since it may be necessary to understand what data are available before attempting to develop SEPs and characterizing the duration, frequency, and intensity of exposures. Further, different techniques are likely to be needed to consider and fill data gaps, and important information such as the determinants of exposure may become more central to the exposure assessment (i.e., the conditions of exposure, such as the presence of ventilation or other exposure controls, employee work practices, and the nature and location of exposures such as indoors vs. outdoors) (18). Finally, while variability and uncertainty may be greater than ideal or preferred, the reporting and validation steps remain critical to the conclusions and presentation of the results of the assessment. Also in relation to both retrospective and prospective assessments, although repeatability is important, there are certain scenarios where results may vary due to limited data availability and the impact of subjective decisions made throughout the assessment process. These potential differences emphasize the importance of reassessment and review throughout the exposure assessment.

#### Aggregate, cumulative, and comparative exposure assessments

Additional contexts in which the UEAF structure may require adjustment are aggregate and cumulative exposure assessments, as well as comparative exposure assessments. Aggregate exposure assessments integrate multiple routes and pathways for a single substance, whereas cumulative exposure assessments consider combined exposures to multiple stressors that may share common health outcomes or mechanisms of action. Both approaches require expanded coordination across framework steps and rely on harmonized assumptions about temporal and spatial exposure patterns, route-specific determinants, and overlapping populations (3, 7, 55, 56). Because these assessments evaluate exposures across multiple pathways or stressors, iteration between exposure characterization (Steps 2 and 3) and variability and uncertainty evaluation (Steps 5 and 6) becomes especially important to ensure internal consistency. Teeguarden et al. emphasized that the aggregate exposure pathway (AEP) construct provides an organizational model for aligning data and models across routes, reinforcing that multi-pathway integration can be accommodated without altering the fundamental logic of the UEAF (31). This emphasis on integrating exposures across pathways is consistent with guidance that exposure characterization should reflect the overall decision context and account for variability across the full exposure scenario rather than evaluating pathways in isolation.

By contrast, comparative exposure assessments, such as those described in NRC and the EPA’s Comparative Risk Framework Methodology (CRFM), require modifications in both emphasis and interpretation (6, 57). In these applications, the framework’s process steps remain intact, but the analytical focus shifts toward evaluating relative differences among alternatives, interventions, or management options rather than absolute exposure magnitude. The CRFM illustrates how integrating risk assessment with cost-effectiveness and common-metric approaches (e.g., Quality-Adjusted Life Years) supports transparent, structured comparison of disparate health risks (57). Within the UEAF, this implies an expanded role for early scoping (Step 1) to clearly define the basis for comparison, and for later steps (e.g., Step 5–6) to rigorously address uncertainty propagation and model structure differences, as underscored by EPA and NRC (6, 57). Thus, while the UEAF sequence may still be applied, aggregate or comparative contexts may place different emphasis on particular analytical steps depending on the assessment objective.

Across aggregate, cumulative, and comparative exposure applications, the UEAF may provide a useful organizing structure while allowing some adaptation to the assessment context. The emphasis on harmonization of data sources, temporal boundaries, assumptions, and analytical methods is essential to ensure that results remain comparable and scientifically transparent across pathways, routes, or alternatives. As shown in Figure 1, reassessment and iteration are included in the process, allowing the framework to adapt as information evolves without compromising its foundational sequence. Whether applied to multi-route exposures or evaluations of alternative scenarios, the UEAF provides a harmonized, stepwise approach for organizing key exposure assessment elements across occupational and environmental human health exposure contexts.

## Conclusion

Through this review of the identified U.S. human health exposure assessment frameworks, it was determined that the terminology for the various steps differed and some steps may have been excluded from certain frameworks, but the underlying basis for the steps that were included in the individual frameworks were similar and consistent across nearly all identified comprehensive frameworks. This review condenses the steps across the identified frameworks into one succinct framework with questions and considerations that fall under each step to ensure a comprehensive and consistent exposure assessment approach. Through the establishment of this Unified Exposure Assessment Framework (UEAF) for human health risk assessment, all steps within each of the identified relevant U.S. frameworks are accounted for and reflected in Table 1. Although some frameworks did not explicitly include every UEAF step (as shown in Table 1), the consolidated framework captures the principal exposure assessment elements identified across the reviewed sources. Although the technical details to be performed for each step of the UEAF are not presented in this paper, the unification of the identified U.S. exposure assessment approaches ensures a practitioner’s exposure assessment includes all elements necessary to best inform decision-making based on its results. Rather than replacing existing detailed guidance, the UEAF is intended to help OEHS and exposure science practitioners organize and document exposure assessment activities in a more consistent and transparent manner across occupational and environmental contexts. Future efforts are recommended to compare and harmonize the existing guidance on specific sub-topics within the exposure assessment framework such as defining SEPs and statistical approaches.
