# Supplementary material for: Review of human health exposure assessment frameworks in the United States and development of a unified framework for exposure assessment practitioners
Source: Front Public Health. 2026 Sep 29;14:1860994. doi: 10.3389/fpubh.2026.1860994 (PMC13660188; doi:10.3389/fpubh.2026.1860994)
Supplement: Supplementary file 1 [file Data_sheet_1.pdf]

Supplementary Reference List

From review of key U.S. regulatory bodies, consensus guidance documents, and peer-reviewed scientific articles and book chapters, 99 documents and reports were reviewed. Please see below for the full list of documents, reports and web pages. This list includes references reflected in the paper.

1. ACGIH. *Modern Industrial Hygiene: Recognition and Evaluation of Chemical Agents*. 2nd ed. Cincinnati, Ohio: ACGIH. (2008).

2. AIHA. Introduction to Occupational Exposure Bands (OEBs) Guidance Document. Falls Church, VA: AIHA Exposure and Control Banding Committee (2021)

3. AIHA. Technical Framework: A One-of-a-Kind Resource for all IAQ/IEQ Practitioners. Falls Church, VA: AIHA. (2023)

4. AIHA. 2024. Technical Framework: Guiding Principles for Exposure Risk Assessment and Management. Falls Church, VA: AIHA. (2024)

5. AIHA Guideline Foundation. Principles of Good Practice, Version 5. Falls Church, VA: AIHA. (2024)

6. AIHA Guideline Foundation. Emergency Response Planning Guidelines. Falls Church, VA: AIHA. (2026)

7. Armstrong, T. W., R. T. Zaleski, W. J. Konkel, and T. J. Parkerton. A tiered approach to assessing children's exposure: a review of methods and data. *Toxicol Lett.* (2002) 127:111–9. doi: 10.1016/S0378-4274(01)00490-8

8. Arnone, M., D. Koppisch, T. Smola, S. Gabriel, K. Verbist, and R. Visser. Hazard banding in compliance with the new Globally Harmonised System (GHS) for use in control banding tools. *Regul Toxicol Pharmacol.* (2015) 73:287–95. doi: 10.1016/j.yrtph.2015.07.014

9. Asante-Duah, K. Chapter 7: Principal Elements of a Public Health Risk Assessment for Chemical Exposure Problems, and Chapter 9: Exposure Assessment: Analysis of Human Intake of Chemicals. In: Asante-Duah, K, editor. *Public Health Risk Assessment for Human Exposure to Chemicals*. 2nd ed. Netherlands: Springer Dordrecht (2017) p. 149-167 and 189-229

10. ATSDR. Exposure Dose Reconstruction Program. US Department of Health and Human Services; 2007 [accessed: 2026 Feb]. Available online at: https://stacks.cdc.gov/view/cdc/27153.

11. ATSDR. Exposure Assessment Protocol: Biological and Environmental Sampling of Per- and Polyfluoroalkyl Substances (PFAS). In Protocol, PFAS Exposure Assessment, v3.0. Atlanta, GA: ATSDR. (2020)

12. ATSDR. Public Health Assessment Guidance Manual. US Department of Health and Human Services; 2022 [accessed: 2026 Feb]. Available online at: https://www.atsdr.cdc.gov/pha-guidance/index.html.

13. ATSDR. Assessment of Chemical Exposures (ACE) Program. US Department of Health and Human Services; 2025 [accessed: 2026 Feb]. Available online at: https://atsdr.cdc.gov/ace/php/about/index.html#cdc_program_profile_resources-resources.

14. ATSDR. Public Health Assessment Training (PHAT) Modules: Module 4 - Exposure Pathway Evaluation. US Department of Health and Human Services; 2026 [accessed: 2026 Feb]. Available online at: https://www.atsdr.cdc.gov/pha-training/php/training/exposure-pathway-evaluation.html.

15. Banerjee, S., G. Ramachandran, M. Vadali, and J. Sahmel. Bayesian hierarchical framework for occupational hygiene decision making. *Ann Occ Hyg.* (2014) 58:1079-93. doi: 10.1093/annhyg/meu060

16. Benke, G., M. Sim, A. Forbes, and M. Salzberg. Retrospective assessment of occupational exposure to chemicals in community-based studies: validity and repeatability of industrial hygiene panel ratings. *Int J Epidemiol.* (1997) 26:635-42. doi: 10.1093/ije/26.3.635

17. Boutin, B., Brown, M.B., Clark, R., Lipscomb, J.C., Miltner, R., Murphy, P., Papa, L.R., Reasoner, D., and Rheingans, R. *Comparative Risk Framework Methodology And Case Study*. Washington, DC: U.S. Environmental Protection Agency, Office of Research and Development, National Center for Environmental Assessment, Washington Office. (1998)

18. CDC. National Biomonitoring Program. US Department of Health and Human Services; 2024 [accessed: 2026 Feb]. Available online at: https://www.cdc.gov/biomonitoring/about/index.html.

19. Charleston, A. E., H. R. Wilson, P. O. Edwards, F. David, and S. Dewitt. Environmental Public Health Tracking: Driving Environmental Health Information. *J Public Health Manag Pract*. (2015) 21:S4-S11. doi: 10.1097/PHH.0000000000000173

20. Chronic Hazard Advisory Panel on Phthalates. Report to the US Consumer Product Safety Commission by the Chronic Hazard Advisory Panel on Phthalates and Phthalate Alternatives. (2014)

21. Cohen Hubal, E. A., J. J. Frank, R. Nachman, M. Angrish, N. C. Deziel, M. Fry, R. Tornero-Velez, A. Kraft, and E. Lavoie. Advancing systematic-review methodology in exposure science for environmental health decision making. *J Exp Sci Environ Epidemiol*. (2020) 30:906-916. doi: 10.1038/s41370-020-0236-0

22. Cohen Hubal, E. A., L. S. Sheldon, J. M. Burke, T. R. McCurdy, M. R. Berry, M. L. Rigas, V. G. Zartarian, and N. C. Freeman. Children's exposure assessment: a review of factors influencing Children's exposure, and the data available to characterize and assess that exposure. *Environ Health Perspect*. (2000) 108:475-86. doi: 10.1289/ehp.108-1638158

23. Connolly, A., P. Scheepers, M. Coggins, T. Vermeire, M. van Tongeren, G. Heinemeyer, J. Bridges, S. Bredendiek-Kämper, Y. Bruinen de Bruin, A. Clayson, J. Gerding, J. McCourt, J. Urbanus, S. Viegas, N. Goetz, M. Zare-Jeddi, and P. Fantke. Framework for developing an exposure science curriculum as part of the European Exposure Science Strategy 2020–2030. *Environ Int.* (2022) 168:107477. doi: 10.1016/j.envint.2022.107477

24. CPSC. The Inside Story: A Guide to Indoor Air Quality. US Consumer Product Safety Commission; n.d. [accessed: 2026 Feb]. Available online at: https://www.cpsc.gov/safety-education/safety-guides/home/inside-story-guide-indoor-air-quality.

25. Daston, G., E. Faustman, G. Ginsberg, P. Fenner-Crisp, S. Olin, B. Sonawane, J. Bruckner, W. Breslin, and T. J. McLaughlin. A framework for assessing risks to children from exposure to environmental agents. *Environ Health Perspect*. (2004) 112:238-56. doi: 10.1289/ehp.6182

26. Dourson, M., R. A. Becker, L. T. Haber, L. H. Pottenger, T. Bredfeldt, and P. A. Fenner-Crisp. Advancing human health risk assessment: integrating recent advisory committee recommendations. *Crit Rev Toxicol*. (2013) 43:467–92. doi: 10.3109/10408444.2013.807223

27. Egeghy, P. P., L. S. Sheldon, K. K. Isaacs, H. Özkaynak, M.-R. Goldsmith, J. F. Wambaugh, R. S. Judson, and T. J. Buckley. Computational exposure science: an emerging discipline to support 21st-century risk assessment. *Environ Health Perspect*. (2016) 124:697–702. doi: 10.1289/ehp.1509748

28. Fantke, P., N. Von Goetz, U. Schlüter, J. Bessems, A. Connolly, T. Dudzina, A. Ahrens, J. Bridges, M. A. Coggins, and A. Conrad. Building a European exposure science strategy. *J Expo Sci Environ Epidemiol*. (2020) 30:917–24. doi: 10.1038/s41370-019-0193-7

29. Ferguson, A., R. Penney, and H. Solo-Gabriele. A Review of the Field on Children's Exposure to Environmental Contaminants: A Risk Assessment Approach. *Int J Environ Res Public Health*. (2017) 14. doi: 10.3390/ijerph14030265

30. Finkel, A. M., D. O. Johns, and C. Whittaker. Chapter 12: Occupational Risk Assessment. In: M. G. Robson, W. A. Toscano, Q. Meng and D. A. Kaden, editors. *Risk assessment for environmental health*. CRC Press (2022).

31. Flinders, C., B. Barnhart, E. B. Morrison, P. D. Anderson, and W. G. Landis. Probabilistic approaches for risk assessment and regulatory criteria development: current applications, gaps, and opportunities. *Integr Environ Assess Manag*. (2025) 21 (6):1281-1292. doi: 10.1093/inteam/vjaf016

32. Ge, C. B., M. C. Friesen, H. Kromhout, S. Peters, N. Rothman, Q. Lan, and R. Vermeulen. Use and Reliability of Exposure Assessment Methods in Occupational Case–Control Studies in the General Population: Past, Present, and Future. *Annal Work Expos Health*. (2018) 62:1047–63. doi: 10.1093/annweh/wxy080

33. ISES. In: N. v. G. Y. Bruinen de Bruin, U. Schlüter, J. Bessems, A. Connolly, T. Dudzina, A. Ahrens, J. Bridges, M. Coggins, A. Conrad, A. Crépet, G. Heinemeyer, O. Hänninen, S. Kephalopoulos, M. McLachlan, T. Meijster, V. Poulsen, D. Rother, T. Vermeire, S. Viegas, J.

Vlaanderen, M. Zare Jeddi, P. Fantke. *Assessment of Needs for a European Strategy on Exposure Science*. Washington, D.C.: ISES Europe Report. (2019)

34. Jahn, S. D., W. H. Bullock, and J. S. Ignacio. *A Strategy for Assessing and Managing Occupational Exposures*. 4th ed. ISBN: 1935082469. Fairfax, VA: AIHA. (2015)

35. Jayjock, M. A., C. F. Chaisson, S. Arnold, and E. J. Dederick. Modeling framework for human exposure assessment. *J Expo Sci Environ Epidemiol*. (2007) 17:S81–9. doi: 10.1038/sj.jes.7500580

36. Krewski, D., P. Saunders-Hastings, R. A. Baan, T. S. Barton-Maclaren, P. Browne, W. A. Chiu, M. Gwinn, T. Hartung, A. D. Kraft, and J. Lam. Development of an evidence-based risk assessment framework. *Altex*. (2022) 39:667-93. doi: 10.14573/altex.2004041

37. Lentz, T. J., G. S. Dotson, P. R. Williams, A. Maier, B. Gadagbui, S. P. Pandalai, A. Lamba, F. Hearl, and M. Mumtaz. Aggregate Exposure and Cumulative Risk Assessment--Integrating Occupational and Non-occupational Risk Factors. *J Occup Environ Hyg*. (2015) 12(sup1):S112-26. doi: 10.1080/15459624.2015.1060326

38. Liang, D. 2022. Chapter 4: Exposure Assessment. In: M. G. Robson, W. A. Toscano, Q. Meng and D. A. Kaden, editors. *Risk assessment for environmental health*. CRC Press (2022).

39. Lioy, P. J. Exposure science: a view of the past and milestones for the future. *Environ Health Perspect*. (2010) 118:1081–90. doi: 10.1289/ehp.0901634

40. Massarsky, A., E. S. Fung, V. J. Evans, and A. Maier. In silico occupational exposure banding framework for data poor compounds in biotechnology. *Toxicol Ind Health*. (2025) 41:20–31. doi: 10.1177/07482337241289184

41. Meeker, J. D. Chapter 8: Occupational and Environmental Hygiene. In: Levy BS, Wegman DH, Baron SL, and Sokas RK, editors. *Occupational and Environmental Health*. Oxford University Press. (2017)

42. NAS (National Academy of Sciences). *Using 21st Century Science to Improve Risk-Related Evaluations*. Washington, D.C.: National Academies Press (2017).

43. National Research Council (NRC). *Risk Assessment in the Federal Government: Managing the Process*. Washington, DC: The National Academies Press (1983).

44. National Research Council (NRC). *Science and Decisions: Advancing Risk Assessment*. Washington, D.C.: National Academies Press-National Research Council (NRC), Committee on Improving Risk Analysis Approaches, Board of Environmental Studies and Toxicology, Division on Earth and Life Studies (2009).

45. National Research Council (NRC). *Exposure Science in the 21st Century: A Vision and a Strategy*. Washington, D.C.: The National Academies Press (2012).

46. National Research Council (NRC). A *Framework to Guide Selection of Chemical Alternatives*. Washington, D.C.: The National Academies Press (2014).

47. Needham, L. L., H. Ozkaynak, R. M. Whyatt, D. B. Barr, R. Y. Wang, L. Naeher, G. Akland, T. Bahadori, A. Bradman, R. Fortmann, L. J. Liu, M. Morandi, M. K. O'Rourke, K. Thomas, J. Quackenboss, P. B. Ryan, and V. Zartarian. Exposure assessment in the National Children's Study: introduction. *Environ Health Perspect*. (2005) 113:1076–82. doi: 10.1289/ehp.7613

48. Needham, L. L., and K. Sexton. 2000. Assessing children's exposure to hazardous environmental chemicals: an overview of selected research challenges and complexities. *J Expo Anal Environ Epidemiol*. (2000) 10:611–29. doi: 10.1038/sj.jea.7500142

49. Nicas, M., and M. Jayjock. Uncertainty in exposure estimates made by modeling versus monitoring. *AIHA J*. (2002) 63:275-83. doi: 10.1080/15428110208984714

50. Nieuwenhuijsen, M. J. Chapter 1: Introduction to Exposure Assessment. In: Nieuwenhuijsen, M. J, editor. *Exposure assessment in environmental epidemiology.* Oxford University Press (2015).

51. NIOSH. In: Leidel NA, Busch KA and Lynch JR, editors. *Occupational Exposure Sampling Strategy Manual. HEW Publication No. 77–173*. Cincinnati, OH: U.S. Dept. of Health, Education, and Welfare, Public Health Service, Center for Disease Control, National Institute for Occupational Safety and Health (1977).

52. NIOSH. *Qualitative Risk Characterization and Management of Occupational Hazards: Control Banding (CB) - A Literature Review and Critical Analysis*. Department of Health and Human Services (2009).

53. NIOSH. *Fundamentals of Total Worker Health Approaches: Essential Elements for Advancing Worker Safety, Health, and Well-Being*. In: Lee MP, Hudson H, Richards R, Chang CC, Chosewood LC, Schill AL, on behalf of the NIOSH Office for Total Worker Health. Cincinnati, OH: U.S. Department of Health and Human Services, Centers for Disease Control and Prevention, National Institute for Occupational Safety and Health, DHHS (NIOSH) Publication No. 2017-112. (2016).

54. NIOSH. *Technical Report: The NIOSH Occupational Exposure Banding Process for Chemical Risk Management*. Department of Health and Human Services (2019).

55. NIOSH. "Current intelligence bulletin 69: NIOSH practices in occupational risk assessment". In: Daniels RD, Kuppusamy SP, Kuempel ED, Park RM, Pandalai SP and Smith RJet al, editors. *DHHS (NIOSH) Publication No.: 2020–106. Revised March, 2020*. Cincinnati, OH: U.S. Dept. of Health and Human Services--Centers for Disease Control and Prevention--National Institute for Occupational Safety and Health (NIOSH) (2020a)

56. NIOSH.. In: Andrews R and O'Connor PF, editors. *NIOSH Manual of Analytical Methods (NMAM), 5th Edition.* US Department of Health and Human Services, Public Health Service, Centers for Disease Control (2020b)

57. NIOSH. 2021. Health Hazard Evaluation Program. National Institute of Occupational Safety and Health; 2026 [accessed: 2026 Feb]. Available online at: https://www.cdc.gov/niosh/hhe/about/index.html

58. NIOSH. Exposure Assessment Program; 2024 [accessed: 2026 Feb]. Available online at: https://www.cdc.gov/niosh/research-programs/portfolio/exposure-assess.html.

59. NIOSH. Skin Exposures and Effects; 2026 [accessed: 2026 Feb]. Available online at: https://www.cdc.gov/niosh/skin-exposure/about/index.html.

60. OSHA. Recommended Practices for Safety and Health Programs: Hazard Identification and Assessment. 2016 [accessed: 2026 Feb]. Available online at: https://www.osha.gov/safety-management/hazard-Identification#ai6.

61. OSHA. *OSHA Technical Manual: Section II, Chapter 1 Personal Sampling for Air Contaminants*. Washington, D.C.: OSHA (Occupational Safety and Health Administration) (2023).

62. OSHA. *OSHA Field Safety and Health Management System (SHMS) Manual: Chapter 27. Exposure Monitoring.* Occupational Safety and Health Administration; n.d. [accessed: 2026 Feb]. Available online at: https://www.osha.gov/shms/chapter-27.

63. Ott, W. R. 1985. Total human exposure. *Environ Sci Tech*. (1985) 19:880-86. doi: 10.1021/es00140a001

64. Paustenbach, D., Madl, A.K., and Massarsky, A. "Exposure Assessment". In: Paustenbach D, editor. *Human and Ecological Risk Assessment: Theory and Practice*, vol. 1, 3rd Edn. Hoboken, NJ: John Wiley & Sons, Inc. (2024).

65. Paustenbach, D. J. The practice of exposure assessment: a state-of-the-art review. *J Toxicol Environ Health B Crit Rev*. (2000) 3:179–291. doi: 10.1080/10937400050045264

66. Peretz, C., A. Goren, T. Smid, and H. Kromhout. Application of Mixed-effects Models for Exposure Assessment. *Ann Occup Hyg*. (2002) 46:69–77. doi: 10.1093/annhyg/mef009

67. Reiss, R., E. L. Anderson, and J. Lape. A framework and case study for exposure assessment in the Voluntary Children's Chemical Evaluation Program. *Risk Anal*. (2003) 23:1069–84. doi: 10.1111/1539-6924.00383

68. Sahmel, J., K. Devlin, D. Paustenbach, D. Hollins, and S. Gaffney. The role of exposure reconstruction in occupational human health risk assessment: Current methods and a recommended framework. *Crit Rev Toxicol*. (2010) 40:799–843. doi: 10.3109/10408444.2010.501052

69. Seixas, N. S., and H. Checkoway. Exposure assessment in industry specific retrospective occupational epidemiology studies. *Occup Environ Med*. (1995) 52:625–33. doi: 10.1136/oem.52.10.625

70. Sexton, K., M. A. Callahan, and E. F. Bryan. Estimating exposure and dose to characterize health risks: the role of human tissue monitoring in exposure assessment. *Environ Health Perspect*. (1995) 103:13–29. doi: 10.1289/ehp.95103s313

71. Simon, T. Chapter 4: Exposure Assessment. In: Simon T., editor. *Environmental risk assessment: a toxicological approach*. CRC Press (2019).

72. Sussman, R. G., A. R. Schatz, T. A. Kimmel, A. Ader, B. D. Naumann, and P. A. Weideman. Identifying and assessing highly hazardous drugs within quality risk management programs. *Regul Toxicol Pharmacol*. (2016) 79:S11–8. doi: 10.1016/j.yrtph.2016.05.025

73. Teeguarden, J. G., Tan, Y., Edwards, S.W., Leonard, J.A., Anderson, K.A., Corley, R.A., Kile, M.L., Simonich, S.M., Stone, D., Tanguay, R.L., Waters, K.M., Harper, S.L., and Williams, D.E. 2016. Completing the Link between Exposure Science and Toxicology for Improved Environmental Health Decision Making: The Aggregate Exposure Pathway Framework. *Environ Sci Technol*. (2016) 50:4579–86. doi: 10.1021/acs.est.5b05311

74. Tornero-Velez, R., E. Symanski, H. Kromhout, R. C. Yu, and S. M. Rappaport. 1997. Compliance versus risk in assessing occupational exposures. *Risk Anal*. (1997) 17:279-92. doi: 10.1111/j.1539-6924.1997.tb00866.x

75. USDOD. DOEHRS-IH & the 8-Step DoD Exposure Assessment Model. US Department of Defense; 2023 [accessed: 2026 Feb]. Available online at: https://ph.health.mil/topics/workplacehealth/ih/Pages/DOEHRS-IH.aspx.

76. USDOE. DOE Handbook: Environmental Radiological Effluent Monitoring and Environmental Surveillance. In: US Department of Energy, editor. *DOE-HDBK-1216-2015*. Washington, D.C (2022).

77. USEPA. *Risk Assessment Guidance for Superfund. Volume I: Human Health Evaluation Manual (Part A). Interim Final. EPA/540/1-89/002*. Washington, D.C.: Office of Emergency and Remedial Response, U.S. Environmental Protection Agency (1989).

78. USEPA. Guidelines for exposure assessment; notice. *Fed. Regist*. (1992) 57: 22888-938.

79. USEPA. *Framework for Cumulative Risk Assessment. EPA/600/P-02/001F*. Washington, DC: U.S. Environmental Protection Agency, Office of the Research and Development, Center for Public Health and Environmental Assessment (CPHEA), formerly known as the National Center for Environmental Assessment (NCEA) (2003).

80. USEPA. *A Framework for Assessing Health Risks of Environmental Exposures to Children. EPA/600/R-05/093F*. Washington, DC: US Environmental Protection Agency, National Center for Environmental Assessment (2006).

81. USEPA. *Exposure Factors Handbook, 2011 Edition. EPA/600/R-090/052F 2011*. Washington, D.C.: U.S. Environmental Protection Agency. National Center for Environmental Assessment, Office of Research and Development (2011).

82. USEPA. *Standard Operating Procedure for Residential Pesticide Exposure Assessment*. Washington, D.C.: Health Effects Division, Office of Pesticide Programs, Office of Chemical Safety and Pollution Prevention, U.S. Environmental Protection Agency (2012).

83. USEPA. *Framework for Human Health Risk Assessment to Inform Decision Making. EPA/100/R-14/001*. Washington, D.C.: U.S. Environmental Protection Agency, Office of the Science Advisor, Risk Assessment Forum (2014).

84. USEPA. *Guidelines for Human Exposure Assessment. EPA Document #EPA/100/B-19/001*. Washington, D.C.: U.S. Environmental Protection Agency (EPA) (2019).

85. USEPA. *Draft Systematic Review Protocol Supporting TSCA Risk Evaluations for Chemical Substances Version 1.0. A Generic TSCA Systematic Review Protocol with Chemical-Specific Methodologies. EPA Document #EPA-D-20-031*. Washington, DC: U.S. Environmental Protection Agency (EPA) (2021).

86. USEPA. *Guidelines for Cumulative Risk Assessment Planning and Problem Formulation. EPA/100/B-24/001*. Washington, D.C.: Risk Assessment Forum (2025a).

87. USEPA. Integrated Risk Information System (IRIS). US Environmental Protection Agency; 2025b [accessed: 2026 Feb]. Available online at: https://assessments.epa.gov/risk/document/&deid%3D2776.

88. USFDA. *Preparation of Food Contact Notifications for Food Contact Substances (Toxicology Recommendations): Guidance for Industry*. Washington D.C.: US Department of Health and Human Services, Food and Drug Administration, Center for Food Safety and Applied Nutrition (2021).

89. USFDA. Cumulative Estimated Daily Intake (CEDI) Database. 2026a [accessed: 2026 Feb]. Available online at: https://hfpappexternal.fda.gov/scripts/fdcc/index.cfm?set=CEDI.

90. USFDA. Expanded Decision Tree: FDA's Food Chemical Toxicity Screening Tool. 2026b [accessed: 2026 Feb]. Available online at: https://www.fda.gov/food/food-chemical-safety/expanded-decision-tree-fdas-food-chemical-toxicity-screening-tool.

91. USFDA. FDA Total Diet Study (TDS). 2026c [accessed: 2026 Feb]. Available online at: https://www.fda.gov/food/reference-databases-and-monitoring-programs-food/fda-total-diet-study-tds.

92. Viet, S. M., J. C. Falman, L. S. Merrill, E. M. Faustman, D. A. Savitz, N. Mervish, D. B. Barr, L. A. Peterson, R. Wright, and D. Balshaw. Human Health Exposure Analysis Resource (HHEAR): A model for incorporating the exposome into health studies. *Int J Hyg Environ Health*. (2021) 235:113768. doi: 10.1016/j.ijheh.2021.113768

93. Viet, S. M., M. Stenzel, C. Rennix, T. Armstrong, and J. Couch. *Guideline on Occupational Exposure Reconstruction*. 1st ed. Fairfax, VA: American Industrial Hygiene Association Press (2008).

94. Wallace, L. A. *The Total Exposure Assessment Methodology (TEAM) Study: Summary and Analysis, Volume I. EPA/600/6-87/002a*. Washington, D.C.: U.S. Environmental Protection Agency, Office of Research and Development (1987).

95. Weaver, V. M., T. J. Buckley, and J. D. Groopman. Approaches to environmental exposure assessment in children. *Environ Health Perspect*. (1998) 106:827–32. doi: 10.1289/ehp.98106827

96. WHO. *Environmental Health Criteria 214: Human Exposure Assessment*. Geneva, Switzerland: World Health Organization (2000).

97. Zalk, D. M., and D. I. Nelson. 2008. History and evolution of control banding: a review. *J Occup Environ Hyg*. (2008) 5:330–46. doi: 10.1080/15459620801997916

98. Zartarian, V., T. Bahadori, and T. McKone. 2005. Adoption of an official ISEA glossary. *J Exp Sci Environ Epidemiol.* (2005) 15:1-5. doi: 10.1038/sj.jea.7500411

99. Zartarian, V., W. Ott, and N. Duan. Chapter 2: Basic Concepts and Definitions of Exposure and Dose. In: W. R. Ott, Steinemann, A.C., and Wallace, LA, editors. *Exposure analysis*. CRC Press (2006).
